# Relationship between psychological health and quality of life of people affected by leprosy in the community in Guangdong province, China: a cross-sectional study

**DOI:** 10.1186/s12889-019-6672-x

**Published:** 2019-04-23

**Authors:** Mingzhou Xiong, Xiaohua Wang, Ting Su, Bin Yang, Ming Li, Daocheng Zheng

**Affiliations:** 1 Leprosy Control Department, Dermatology Hospital of Southern Medical University, Guangdong Province, Guangzhou, China; 2 Leprosy Control Department, Guangdong Provincial Center for Skin Disease and STI Control, Guangdong Province, Guangzhou, China

**Keywords:** Leprosy, Psychological health, Quality of life, Cross-sectional study

## Abstract

**Background:**

This paper investigates the relationship between psychological health and quality of life (QOL) of people affected by leprosy (PAL) living in a community in Guangdong province, China.

**Methods:**

A population-based cross-sectional survey was conducted from April to October 2016 in Guangdong province, China. The 12-item general health questionnaire (GHQ12) and World Health Organization Quality of life-BREF (WHOQOL-BREF) were used to evaluate the psychological health and QOL of the participants. PAL recruited for this study included those who were (1) registered in LEPMIS by the end of 2015 and living in the community, (2) able to be contacted by investigators, and (3) willing to provide informed written consent for enrolling in the study. Proportions, medians, and ratios were used to describe the demographics of the participants and χ2 test was used to compare groups with different psychological health states. Pearson’s correlation coefficient and logistic regression analyses were used to assess the relationship between GHQ12 and QOL score.

**Results:**

A total of 7230 PAL (5527 males and 1701 females, average age 67.3 ± 13.1 years) living in the community participated in the study. The participants averaged 1.7 ± 2.7 points on the GHQ12. Of these, 23.5% had scores meeting a psychological disorder (≥3 points). The scores for the physical, psychological, and social relationships, and environment dimensions of QOL were 17.2 ± 2.4, 20.6 ± 2.7, 9.7 ± 1.7, and 24.6 ± 4.0, respectively. Gender, age, employment, profession, and the four dimensions of QOL were independent factors associated with psychological health of PAL.

**Conclusion:**

In addition to focusing on the factors associated with poor QOL and psychological health amongst PAL, there is an urgent need for stigma reduction, rehabilitation programs and social integration. This may be achieved by engaging community members together with PAL to design a locally tailored intervention program.

## Background

Leprosy, also known as Hansen’s disease, is a chronic infectious disease caused by *Mycobacterium leprae* bacteria. Leprosy is characterized by the formation of granulomas in nerves, respiratory tract, skin, and eyes [1, 2]. A total of 174,608 new leprosy cases were reported in 136 countries worldwide in 2015, corresponding to a prevalence rate (PR) of 0.29 per 10,000 individuals [3], which is lower than the PR of 0.32 per 10,000 in 2014 [4]. The Chinese government initiated a ten-year (2011–2020) project in 2011 in an effort to eliminate leprosy. These efforts, along with sustained economic growth, have resulted in a rapid decline in endemic leprosy in China in recent years. The number of new cases of leprosy in 2015 was 678, which was a 40.7% reduction compared to 2011 [5]. This rate of decline was substantially higher than the global average rate and also the average rate from 2001 to 2010 in China [6]. Leprosy can cause an inability to feel pain, and consequently repeated injuries and infections caused by unnoticed wounds, which can result in loss of parts of the extremities. Leprosy can also cause weakness and poor eyesight. While these clinical symptoms and consequent disabilities caused by leprosy can persist for a life time, the stigma attached to this disease often outweighs the physical suffering among people affected by leprosy (PAL).

PAL also refers to those who have completed treatment and have become cured. Unlike in the past when PAL lived in leprosaria, PAL now live with families and friends in their hometown and can work as their physical health allows. In the past, the Chinese government built leprosaria to isolate and shelter PAL. After PAL have become cured, most had come back home. However, some still lived in leprosaria due to discrimination and homelessness in their native area. This subgroup of leprosy patients has been defined as “PAL living in leprosaria”. In the 1980s, multiple drug therapy was popularized in China, and separation became unnecessary for leprosy patients. Treatment can be obtained from local leprosy control institutions, and few patients were sent to leprosaria. Government provided subsidies to PAL living in leprosaria to cover their essential livelihood and medical needs, but no specific subsidies were available for PAL living in the community at large. Discrimination against leprosy is widespread in China. PAL are often ostracized by their communities [7]; this discrimination may come from neighbors, family members, and medical staff [8–10], which can significantly affect the quality of life (QOL) of the PAL [11]. Moreover, physical rehabilitation aids and stigma reduction programs targeting PAL living in community are challenging for several reasons: first, as PAL tend to disperse within local communities, identification of PAL is difficult; second, PAL tend to conceal their conditions to protect themselves from being identified and stigmatized [12]. PAL are vulnerable to economic, psychological, and social pressures. This negatively impacts access to care, treatment outcomes, and social functioning. Discrimination may also result in loss of wages and impede utilization of medical services [13]. Furthermore, discrimination is an important cause of delayed diagnosis, which can facilitate the transmission of leprosy within families and communities [12, 14].

Despite efforts by the Chinese government to decrease the incidence of leprosy and increase treatment accessibility, the psychological and social rehabilitation of PAL living in communities have not been adequately addressed. The psychological status and QOL of PAL living in community tend to be lower than in the general population due to prevailing poor attitudes of society towards leprosy and aggravated by disability caused by leprosy [15–17].Leprosy negatively impacts the physical and social functioning of PAL, which may in turn influence their psychological status [18, 19]. The psychological health of PAL is affected by several economic and social factors and is intimately connected with QOL [20]. Currently, information about PAL and their status is retained by government leprosy control institutions and are not publicly available. Thus far, there have been no large studies in mainland China to explore the QOL of PAL who have completed treatment. The main objective of this study was to investigate the psychological status and QOL of PAL living in community and to explore the relationship between these two factors in a large sample of PAL in China. We conducted a cross-sectional study on PAL in the Guangdong Province.

## Method

### Research context

Guangdong Province is located in southern China. It’s warm and humid climate is a contributing factor to the region’s leprosy endemic [21]. Records show that between 1949 and 2015, 96,184 people in the Guangdong province became infected with leprosy, accounting for approximately 20% of all leprosy patients in China [22]. While the prevalence of leprosy dropped to fewer than 0.1 case per 100,000 people in 2011 in the Guangdong, this province continues to carry a high burden of leprosy in China. In 2017, 74 new cases were detected and 273 leprosy patients were undergoing treatment in Guangdong. Guangdong has established 21 municipal and 98 county level leprosy control institutions, which provide coverage for the whole province. All PAL living in the community at large in Guangdong were registered in the Leprosy Management Information System (LEPMIS) in China, which was established in 2010, and their information including name, gender, age, and family address can be searched from this system.

### Study design

We conducted a population-based cross-sectional survey of PAL living in the community at large in Guangdong Province, China from April to October 2016.

### Study site and participants

This study was conducted in Guangdong Province, and about 21 municipal and 108 county level leprosy institutions collected and submitted their data. PAL who were registered in the LEPMIS were included in this research. Each municipal and county leprosy control institution took charge of local investigations, in which leprosy doctors and healthcare workers served as investigators. The local civil affairs bureau and public security bureau supplied necessary information to help reach PAL.

PAL recruited for this study were those who were (1) registered in LEPMIS by the end of 2015 and living in the community at large, (2) able to be contacted by investigators, and (3) willing to provide written informed consent. Individuals with complications from other severe diseases or those with logopathy or other expression disorders that prevent them from accurately answering survey questions were excluded.

### Participant recruitment and questionnaire administration

The procedure of participant recruitment was as follows:

1. The information of each person was downloaded from LEPMIS;

2. To account for outdated information in LEPMIS, local public security assisted to identity persons who were no longer living, who were excluded from the study.

3. The contact information and family address of PAL were updated with the help of local civil affairs bureau.

4. Using the updated contact information, PAL were sought out using a door-to-door approach. To protect the privacy of PAL, the investigation was integrated into a follow-up visit by the essential public health service program in China.

5. The questionnaire was administered after obtaining the written informed consent from participants. Informed consent included information about study objectives, rights of study participants, and measures to protect privacy and confidentiality. A paper-based questionnaire was used in this survey with questions written in Chinese. Each question was asked by the investigator in Mandarin Chinese and provincial dialect was used if necessary.

### Data collection

A total of 7690 PAL living in community were confirmed to be living and able to be located. Of these, 7230 PAL (5527 males and 1701 females) participated in the study. Among the 460 PAL not participating in the study, 311 worried about their information security, 110 were excluded due to communication problems or sickness from other severe diseases, and the remaining 39 PAL provided invalid questionnaires. Demographics of all participants, including gender, age, education, and profession, as well as psychological status and QOL of the participants were collected. Completed questionnaires were sent to local leprosy control institution and entered into Epidata. The Epidata databases were submitted from county level leprosy control institutions to municipal level leprosy control institutions, and then sent to the research team.

### Measurement

The primary outcome of this study is psychological status of PAL, which was explored using the 12-item general health questionnaire (GHQ12) [23]. The GHQ12 has been previously validated for measurement of psychological disorder [20]. The standard GHQ method of scoring 0–0–1-1 was employed for each item, which allowed a maximum score of 12, and those with a GHQ score of 3 or higher were considered to have a psychological disorder [24]. The WHO Quality of life-BREF (WHOQOL-BREF) scale [25] was applied to evaluate the QOL of the participants. The WHOQOL-BREF has been tested and validated in various cultures under the coordination of the World Health Organization Quality of Life Group. This instrument contains 26 questions, where two are general questions and the remaining 24 are divided into four dimensions: physical (Domain 1, including B3, B4, B10, B15, B16, B17 and B18), psychological (Domain 2, including B5, B6, B7, B11, B19 and B26), social relationships (Domain 3, including B20, B21 and B22) and environmental (Domain 4, including B8, B9, B12, B13, B14, B23, B24 and B25). Each question uses a 5-point response scale (1 = not at all, 2 = a little, 3 = moderately, 4 = mostly, and 5 = completely), and the raw score of each dimension is 7–35, 6–30, 3–15, and 8–40, respectively, with higher scores indicating a better QOL. The psychological dimension of WHOQOL-BREF is one aspect of the QOL and cannot be used to assess psychological status independently. Therefore, we selected GHQ12 to evaluate psychological status. Questionnaires with more than 10% missing data were defined as invalid.

### Statistical analysis

Epidata 3.0 was used to create a database of participant information and all data were double entered and checked for consistency. Data analysis was performed using SPSS V.24.0. Proportions were used to describe the demographics and χ2 test was used to assess statistically significant differences of psychological status among different groups. The central tendency of QOL was described and t-test was used to determine the level of significance among different psychological status groups. Pearson’s correlation coefficient was employed to assess the correlation between the QOL and GHQ12 scores. Then, the GHQ scores were classified based on the cutoff value (< 3 points vs. ≥3 points). Univariable logistic regression was performed to identify potential confounders of GHQ. Multivariable logistic regression analysis was performed to evaluate the relationship between the GHQ12 score and the dimensions of QOL. The GHQ12 score was set as a dependent variable, while the physical, psychological, social relationships, and environment scores were set as independent variables in the logistic regression analysis model. Variables with *p*-value less than 0.10 in the bivariate logistic regression were included in the multivariable logistic regression as covariates, and stepwise method was used for the variable selection strategy of multivariable regression. Both crude odds ratios (OR) and adjusted odds ratios (aOR) with 95% confidence intervals (CI) were reported. Results were considered statistically significant if *p* < 0.05.

### Ethics approval and consent to participate

This research has been reviewed and approved by Ethics Review Committee of Guangdong Provincial Center for Skin Disease and STI Control. Each participant provided written informed consent to participate in the questionnaire and investigation, and all participants below the age of 18 were provided consent by their parents or legal guardians.

## Results

### Demographics

A total of 7230 (5527 male and 1701 female) PAL completed the questionnaire and participated in the study. The average age of the participants was 67.3 ± 13.1 years. Most were more than 50 years old (91.7%) and had primary school education or were illiterate (80.7%). Less than 20% participants had a full- or part-time job and 70.4% defined themselves as unemployed. About 4896 (67.7%) were peasants and 4.7 and 2.3% worked in factories and business, respectively (Table 1).

**Table 1:** Demographic characteristics by psychological health status

		< 3 point *n*(%)	≥3 point *n*(%)	Total *n*(%)	χ2	*P* value
Gender	Male	4302(77.8)	1225(72.1)	5527(76.4)	23.52	< 0.001
	Female	1227(22.2)	474(27.9)	1701(23.5)		
	No data	2(0.1)	0	2(0.1)		
Age	< 30	112(2.0)	16(0.9)	128(1.8)	207.08	< 0.001
	30–49	412(7.4)	62(3.6)	474(6.6)		
	50–69	2736(49.5)	591(34.8)	3327(46.0)		
	≥70	2271(41.1)	1030(60.6)	3301(45.7)		
Education	Primary school or below	4304(77.8)	1531(90.1)	5835(80.7)	126.23	< 0.001
	Junior high school	1015(18.4)	144(8.5)	1159(16.0)		
	Senior high school	155(2.8)	18(1.1)	173(2.4)		
	College and above	50(0.9)	5(0.3)	55(0.8)		
	No data	7(0.1)	1(0.1)	8(0.1)		
Employment	Full-time	964(17.4)	75(4.4)	1039(14.4)	317.89	< 0.001
	Part-time	336(6.1)	36(2.1)	372(5.1)		
	Retired	603(10.9)	100(5.9)	703(9.7)		
	Unemployed	3606(65.2)	1484(87.3)	5090(70.4)		
	No data	22(0.4)	4(0.2)	26(0.4)		
Occupation	Farmers	3741(67.6)	1155(68.0)	4896(67.7)	69.49	< 0.001
	Factory workers	302(5.5)	37(2.2)	339(4.7)		
	Business men	156(2.8)	12(0.7)	168(2.3)		
	Others	1320(23.8)	489(28.7)	1809(25.0)		
	No data	12(0.2)	6(0.4)	18(0.2)		

### QOL score of participants with different psychological health status

The participants averaged 1.7 ± 2.7 points on the GHQ12. Of these, 23.5% scored in range that can translate to a psychological disorder (≥3 points). The average scores of males and females were 1.6 ± 2.6 and 2.0 ± 3.0, respectively. Participants over 69 years old averaged 2.2 ± 3.1 points, which was higher than those in other age groups. Participants who had a primary school level of education or below had a score (List score) higher than other subgroups.

The score of the physical, psychological, social relationships, and environment dimensions of QOL averaged 17.2 ± 2.4, 20.6 ± 2.7, 9.7 ± 1.7, and 24.6 ± 4.0, respectively. The score for each dimension in participants with 3 points or more on the GHQ12 was significantly lower than that in other groups (*P* < 0.05). (Table 2).

**Table 2:** Quality of Life (QOL) scores by psychological health status

	< 3 point($\overline{x}$ ±s)	≥3 point($\overline{x}$ ±s)	Total($\overline{x}$ ±s)	t	*P* value
Physical	21.2 ± 2.5	18.7 ± 2.7	20.6 ± 2.7	36.013	< 0.001
Psychological	17.7 ± 2.1	15.4 ± 2.5	17.2 ± 2.4	38.213	< 0.001
Social relationships	10.0 ± 1.5	8.6 ± 1.8	9.7 ± 1.7	31.980	< 0.001
Environment	25.3 ± 3.7	22.2 ± 4.0	24.6 ± 4.0	30.001	< 0.001

### Logistic regression analysis of GHQ12 score

QOL was correlated with GHQ12 score (*r* = − 0.504, *P* < 0.001). PAL with lower QOL scores in physical health (aOR = 0.83, 95% CI = -0.80–0.85), psychological health (aOR = 0.74, 95% CI = 0.71–0.77), social relationships (aOR = 0.85, 95% CI = 0.80–0.89), and environment (aOR = 0.97, 95% CI = 0.95–0.99) were more likely to have psychological disorders (GHQ12 score ≥ 3; Table 3). Gender, age, employment, and profession were associated with psychological health status of PAL. Males were more likely to have a psychological disorder (aOR = 1.31, 95% CI = 1.13–1.52) compared with females. Meanwhile, PAL who were more than 70 years old (≥70 vs. < 30: aOR = 0.69, 95% CI = 0.60–0.79), worked full-time (full-time vs. unemployment: aOR = 0.36, 95% CI = 0.27–0.48), worked part-time (part-time vs. unemployment: aOR = 0.39, 95% CI = 0.26–0.57), retired (retired vs. unemployment: aOR = 0.38, 95% CI = 0.29–0.50), worked in factory (factory workers vs. peasants: aOR = 0.89, 95% CI = 0.59–1.35), worked in business (businessmen vs. peasants: aOR = 0.85, 95% CI = 0.44–1.65), or worked in other professions (other professions vs. peasants: aOR = 1.35, 95% CI = 1.16–1.57) had a lower risk of having a psychological disorder. (Table 3).

**Table 3:** Factors associated with psychological disorder (GHQ12 score ≥ 3)

Variables	Univariate analyses			Multivariate analyses		
	*aOR*	*95%CI*	*p*	*aOR*	*95%CI*	*p*
Gender						
female	reference					
male	0.74	0.65–0.83	< 0.001	1.31	1.13–1.52	< 0.001
Age						
< 30	reference					
30–49	0.32	0.19–0.54	< 0.001	0.90	0.49–1.65	0.732
50–69	0.33	0.25–0.44	< 0.001	0.77	0.56–1.08	0.131
≥70	0.48	0.42–0.54	< 0.001	0.69	0.60–0.79	< 0.001
Education						
primary school or below	reference					
junior high school	2.51	2.09–3.03	< 0.001	–	–	–
senior high school	3.06	1.93–5.18	0.5342	–	–	–
college and up	3.56	1.56–10.25	0.1871	–	–	–
Employment						
unemployment	reference					
full-time	0.47	0.34–0.64	< 0.001	0.36	0.27–0.48	< 0.001
part-time	0.65	0.43–0.97	0.03	0.39	0.26–0.57	< 0.001
retired	2.48	1.99–3.09	< 0.001	0.38	0.29–0.50	< 0.001
Profession						
peasants	reference					
factory workers	0.40	0.28–0.56	< 0.001	0.89	0.59–1.35	0.587
businessmen	0.25	0.14–0.45	< 0.001	0.85	0.44–1.65	0.624
others^a^	1.20	1.06-1.36	0.004	1.35	1.16–1.57	< 0.001
Quality of life						
physical health	0.68	0.66–0.69	< 0.001	0.83	0.80–0.85	< 0.001
phycological health	0.60	0.58–0.62	< 0.001	0.74	0.71–0.77	< 0.001
social relationship	0.58	0.56–0.60	< 0.001	0.85	0.80–0.89	< 0.001
environment	0.80	0.78–0.81	< 0.001	0.97	0.95–0.99	0.009

^a^others include: teacher, student, driver, office clerk, civil servant, medical staff and unemployed

## Discussion

This study was the first to investigate the psychological health and QOL of PAL living in the community at large in China. Our results showed that PAL living in community had poor psychological health. Also, there was a close association between psychological health and QOL.

The average and distribution of the age of PAL living in a community in Guangdong were similar to those in a study in Zhejiang Province, China, where most PAL were aged 70 (average 69.90 ± 10.65 years old) and lived with chronic diseases (59.86%) [26]. A low education level was also one of the features of PAL in this study, where less than 20% had completed the 9-year compulsory education in China [27]. In this study, psychological disorders were more likely to occur in females, the elderly, and the less educated. The frequency of psychological disorder (23.5%) in PAL in this study was higher than that of general residents in Changchun (21.1%) and Xiamen (18.1%), China [28, 29]. Significant differences were observed between scores for all the dimensions of the QOL between different GHQ12 score groups, where the group with a lower GHQ12 score had a higher QOL score. These results are similar to PAL research conducted in Rio de Janeiro, Brazil and Jharkhand, India [30–32].

Eliminating the hazards of leprosy is an important public health mission in China, where reducing discrimination and promoting QOL in PAL living in community are both part of this mission. Active surveillance and treatment for the wounds of PAL with deformities and disabilities are necessary. In addition, rehabilitation programs for their support and occupational opportunities for them to contribute meaningfully to society will be helpful. In Indonesia, researchers used Rights-Based Counselling to reduce leprosy-related stigma. A significant reduction was found between the before and after total scores of the stigma scale, and the reduction of intervention group was significantly larger than control group (*p* < 0.001) [14]. Self-care groups, which organize PAL together regularly and teach them how to take care of their ulcers and other disabilities, were created in China, Nigeria, and other leprosy endemic countries to promote the physical and psychological rehabilitation of PAL. Some of these programs report that the ulceration prevalence had decreased and family support had increased compared to the control group [33, 34]. Moreover, locally suitable stigma reduction strategies, which aim to increase the social participation of PAL such as in mass meetings, audiovisual shows, short movie, local human resources recruitment or training, can help reduce stigma in a community [35]. Future stigma reduction strategies also should utilize the elements of community engagement, in which community members work together with PAL jointly in the design of tailored stigma reduction interventions for their community [17].

Although the above-mentioned measures presented with promising results, the influence was restricted for these measures were divided as tertiary prevention which aims to promote physical and psychological rehabilitation. More studies are needed to create sustainable interventions of secondary prevention (aims to promote early detection, early diagnosis and early treatment) and primary prevention (aims to reduce pathogenic and risk factor). Leprosy syndromic surveillance program, which integrated the wide coverage of general hospital with the superior leprosy diagnosis ability of leprosy control institution, has been successfully applied in Zhejiang Province to promote leprosy early detection. This program has achieved progress in decreasing disabilities and discrimination, and thus may improve the QOL of leprosy patients and PAL [36]. This model has the potential to be generalized to Guangdong and other districts in China, as well as to be adapted to other leprosy endemic countries.

## Limitations

The marital status of the participants was not obtained due to a mistake in data collection. Most participants were elderly and had low literacy, although one-to-one interviewer-administered survey was used, there still might have been information bias due to poor recall and misunderstanding of the questions. This cross-sectional survey only explored the relationship between QOL and psychological health among PAL. Further studies are needed to identify the causal relationship between QOL and psychological health, or any interaction and moderation effects of covariates.

## Conclusion

In addition to focusing on the factors associated with poor QOL and psychological health amongst PAL, there is an urgent need for stigma reduction, rehabilitation programs and social integration for PAL in China. These may be achieved by engaging community members together with PAL to design locally tailored interventions programs.

## Abbreviations

GHQ12: 12-item general health questionnaire
LEPMIS: Leprosy Management Information System in China
PAL: People affected by leprosy
QOL: Quality of life
SPSS: Statistic Package for Social Science
WHOQOL-BREF: World Health Organization Quality of life-BREF
